# Supplementary material for: Bromoperoxidase Producing Bacillus spp. Isolated from the Hypobranchial Glands of A Muricid Mollusc Are Capable of Tyrian Purple Precursor Biogenesis
Source: Mar Drugs. 2019 May 3;17(5):264. doi: 10.3390/md17050264 (PMC6562550; doi:10.3390/md17050264)
Supplement: Supplementary file 1 [file marinedrugs-17-00264-s001.pdf]

Supplementary Materials:

Bromoperoxidase Producing *Bacillus* spp. Isolated from the Hypobranchial Glands of A Muricid Mollusc Are Capable of Tyrian Purple Precursor Biogenesis

Ajit Kumar Ngangbam [1,†], Peter Mouatt [2], Joshua Smith [2], Daniel L. E. Waters [2,3] and Kirsten Benkendorff [1,*,†]

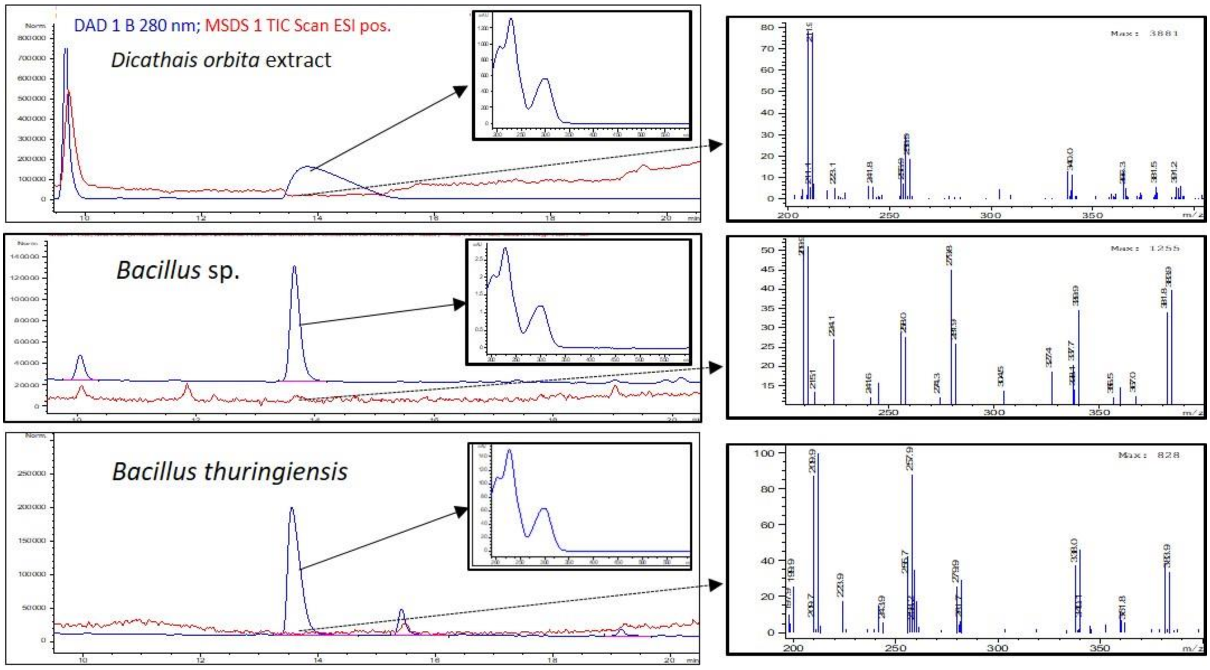

Figure S1: Liquid chromatography-mass spectrometry analysis of dianion resin extracts of *Dicathais orbita* hyprobranchial glands and culture supernatant from two *Bacillus* spp. cultured from the hypobranchial gland. Left panels show the HPLC chromatograms from the diode array detector at 280 nm (blue lines) and total ion current (red lines) in positive ion mode. The UV-Vis spectra is inset for the major peak at 14mins and the right panels show the positive ion mass spectra for the major peaks.

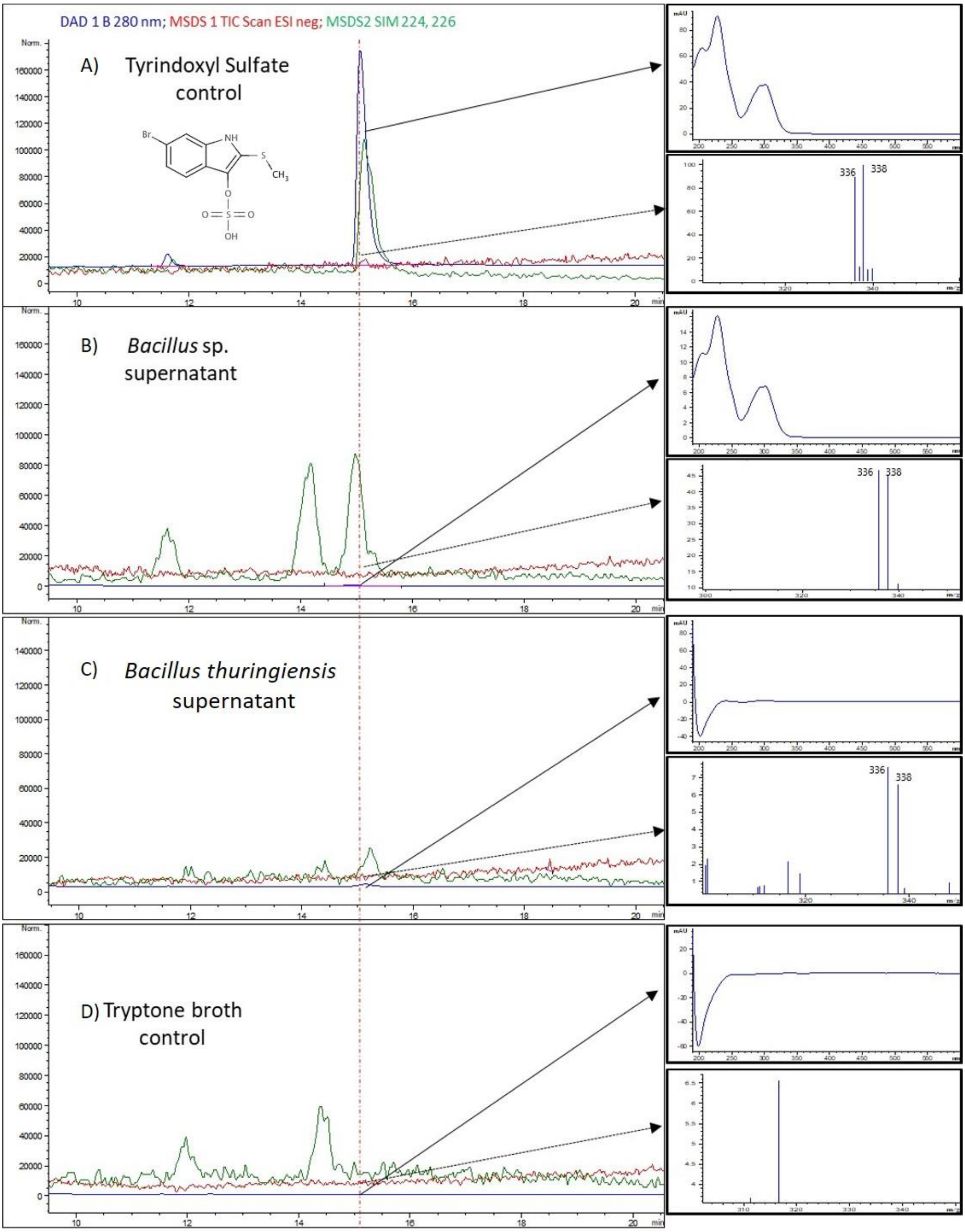

Figure S2. Tyrindoxyl sulfate control (A) and dianion resin extracts from the supernatant of two *Bacillus* species (B, C) cultured from the hypobranchial glands of *Dicthais orbita* and a corresponding tryptone -KBr supplmentaed broth control (D). Left panels show the HPLC scan at 280nm in the diode array (blue), total ion current (TIC) in negative ion mode (red) and selected ion monitoring for major fragment ions at 224, 226 (green). Right panels show the UV Vis spectra and mass spectrum obtained from the apex of the major peak obtained at 15.16 min.
